# A Mobitz type II atrioventricular block in multicentric ischemic stroke

**DOI:** 10.11604/pamj.2016.24.265.8366

**Published:** 2016-07-21

**Authors:** Utku Murat Kalafat, Canan Akman, Turker Karaboga, Tarik Ocak

**Affiliations:** 1 Department of Emergency Medicine, Kanuni Sultan Suleyman Education and Research Hospital, Turkey

**Keywords:** Mobitz Type II AV block, cerebral ischemia, arrhytmia

## Abstract

Cardiac and cerebrovascular illnesses are major causes of mortality and morbidity. Thromboembolisms, which are the result of cardiac arrhythmia, are important causes of ischemic stroke. In this study, we present a rare case of multicentric ischemic stroke induced by Mobitz type II atrioventricular block.

## Introduction

Ischemic stroke is defined as any damage to the central nervous system that is caused by interruption of blood flow. Over 730,000 new stroke cases occur in the United States annually, of which 27% result in death [1]. The reason for ~50% of cardiovascular-based embolic strokes is atrial fibrillation (AF) [2]. However, stroke might cause cardiac arrhythmias such as atrial tachycardia, ventricular tachyarrhythmia, atrioventricular (AV) conduction disturbances, and QT anomalies [3]. Here, we present a multicentric ischemic stroke that was induced by Mobitz type II AV block.

## Patient and observation

**Figure 1:**
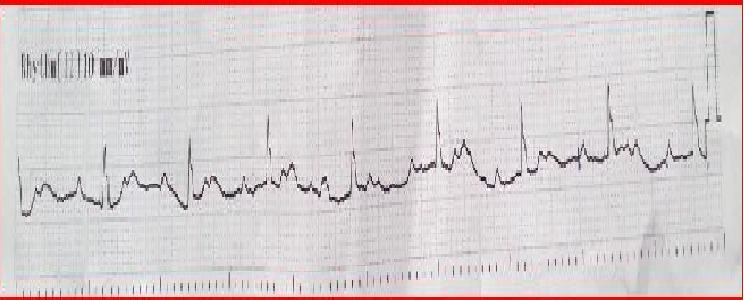
The patient’s EGC rhythm

**Figure 2:**
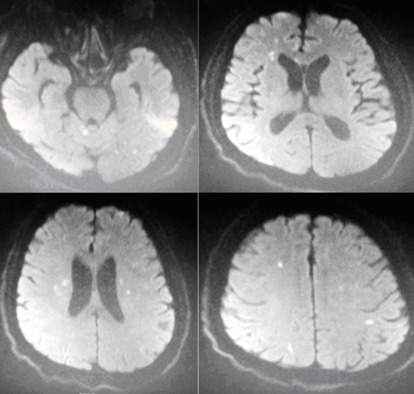
Multicentric acute ischemic hyperdensity of the brain diffusion MRI

A 60-year-old male presented with back pain and lower extremity weakness. He had diabetes mellitus type 2, hypertension, and four lumbar disc hernia. He had been taking antidiabetic and antihypertensive drugs for 5 years, together with anti-inflammatory agents for 1 month. He had a heart rate of 70 beats per minute, a blood pressure of 140/90 mmHg, a fingertip blood sugar of 173 mg/dl, and an oxygen saturation (SpO_2_) of 99%. His electrocardiography (ECG) revealed a normal sinus rhythm. Laboratory analyses revealed that his blood electrolytes were normal. When he was observed in the emergency department, his consciousness and orientation were deteriorated. His vital signs changed to a blood pressure of 120/80 mmHg and a heart rate of 40 beats per minute. His ECG demonstrated a Mobitz type II AV block (Figure 1). The strength in the patient’s lower extremities and Babinski reflex were normal, but deep tendon reflexes were decreased. No acute pathology was detected in a head tomography. Brain diffusion magnetic resonance imaging (MRI) data revealed multiple nodular and linear hyperdensity lesions that were evaluated as acute ischemia (Figure 2). He was hospitalized to the intensive care unit for observation by a neurologist and cardiologist after a transcutaneous cardiac pacemaker had been applied.

## Discussion

Acute strokes can be classified into two general subtypes: ischemic (80-85%) and hemorrhagic 15-20%; [1]. Atherothrombosis, embolism, and hypoperfusion are among the most frequent reasons for ischemic strokes [2]. Iscehmic stroke is often observed together with cardiac arrhythmias such as AF, sick sinus syndrome, and sinus tachycardia. However, a proportion of acute ischemic stroke patients might have cardiac complications such as systolic dysfunction, troponin elevation, AF, or ischemic changes on ECG [4]. Some studies have shown that stroke leads to myocardial damage and increase the incidence of ventricular arrhythmia via a cascade of events that alter the autonomic balance by increasing catecholamine levels [5]. Several conditions including atrial tachycardia, QT anomalies, early repolarization findings, supraventricular tachycardia, and torsades de pointes, which is caused by cerebral ischemia, have been encountered in observed cases [3, 6]. Rem et al. (1985) reported that newly diagnosed arrhythmias were detected in 12 of 184 patients without previous arrhythmias in their history, examination, and admission ECG who were admitted to an Investigative Stroke Unit with transient ischemic attacks and cerebral stroke. Of these, six had AF, four had two degrees of AV block Mobitz type II, as in the current case, and one had each three degrees heart block and sick sinus syndrome [7]. In the current case, Mobitz type II AV block was detected during close observation of the patient who had a multicentric ischemic stroke. Gattringer et al. (2014) examined 421 patients with transient ischemic attacks or ischemic stroke and intracranial hemorrhage who suffered from myocardial infarction during treatment at a stroke unit [8]. Similarly, Wira et al. (2011) supported the importance of cardiac monitoring in patients who present with acute ischemic stroke [4]. We detected Mobitz type II AV block in a fatal arrhythmias using continuous monitoring. We applied a cardiac pacemaker to our patient to rescue the brain tissues from hypoperfusion injury, since the perfusion of brain tissues is very important during the early stages in ischemic stroke patients.

## Conclusion

Stroke patients who are admitted to the emergency department should be observed clinically and undergo continuous cardiac monitoring. We suggest that this positively affects mortality and morbidity by allowing the early diagnosis and treatment of serious heart rhythm abnormalities.
